# Identification of sVSG117 as an Immunodiagnostic Antigen and Evaluation of a Dual-Antigen Lateral Flow Test for the Diagnosis of Human African Trypanosomiasis

**DOI:** 10.1371/journal.pntd.0002976

**Published:** 2014-07-17

**Authors:** Lauren Sullivan, Jennifer Fleming, Lalitha Sastry, Angela Mehlert, Steven J. Wall, Michael A. J. Ferguson

**Affiliations:** 1 Division of Biological Chemistry and Drug Discovery, College of Life Sciences, University of Dundee, Dundee, United Kingdom; 2 BBI Solutions, Alchemy House, Dundee, United Kingdom; New York University School of Medicine, United States of America

## Abstract

**Background:**

The diagnosis of human African trypanosomiasis (HAT) caused by *Trypanosoma brucei gambiense* relies mainly on the Card Agglutination Test for Trypanosomiasis (CATT). There is no immunodiagnostic for HAT caused by *T. b. rhodesiense*. Our principle aim was to develop a prototype lateral flow test that might be an improvement on CATT.

**Methodology/Principle Findings:**

Pools of infection and control sera were screened against four different soluble form variant surface glycoproteins (sVSGs) by ELISA and one, sVSG117, showed particularly strong immunoreactivity to pooled infection sera. Using individual sera, sVSG117 was shown to be able to discriminate between *T. b. gambiense* infection and control sera by both ELISA and lateral flow test. The sVSG117 antigen was subsequently used with a previously described recombinant diagnostic antigen, rISG65, to create a dual-antigen lateral flow test prototype. The latter was used blind in a virtual field trial of 431 randomized infection and control sera from the WHO HAT Specimen Biobank.

**Conclusion/Significance:**

In the virtual field trial, using two positive antigen bands as the criterion for infection, the sVSG117 and rISG65 dual-antigen lateral flow test prototype showed a sensitivity of 97.3% (95% CI: 93.3 to 99.2) and a specificity of 83.3% (95% CI: 76.4 to 88.9) for the detection of *T. b. gambiense* infections. The device was not as good for detecting *T. b. rhodesiense* infections using two positive antigen bands as the criterion for infection, with a sensitivity of 58.9% (95% CI: 44.9 to 71.9) and specificity of 97.3% (95% CI: 90.7 to 99.7). However, using one or both positive antigen band(s) as the criterion for *T. b. rhodesiense* infection improved the sensitivity to 83.9% (95% CI: 71.7 to 92.4) with a specificity of 85.3% (95% CI: 75.3 to 92.4). These results encourage further development of the dual-antigen device for clinical use.

## Author Summary

Human African Trypanosomiasis (HAT) is caused by infection with *Trypanosoma brucei gambiense* or *T. b. rhodesiense*. The diagnosis of *T. b. gambiense* infections currently relies primarily on a Card Agglutination Test for Trypanosomiasis (CATT), which has acknowledged limitations, and there is no simple test for *T. b. rhodesiense* infection. Our overall aim is to produce a simple lateral flow test device with a similar or better sensitivity and specificity than CATT but with better stability and ease of use at point of care. In this study, we identified a particular variant surface glycoprotein, sVSG117, with good diagnostic potential and combined it with a previously identified recombinant diagnostic antigen, rISG65, to produce a prototype dual-antigen lateral flow test. We performed a virtual field trial by testing the device blind with 431 randomized serum samples provided by the WHO HAT Specimen Biobank. The results show that, although the prototype lateral flow test is un-optimized, it was able to diagnose *T. b. gambiense* HAT with a sensitivity and specificity of 97.3% and 83.3% and *T. b. rhodesiense* HAT with a sensitivity and specificity of 83.9% and 85.3%.

## Introduction

Human African Trypanosomiasis (HAT), or African Sleeping Sickness, is caused by two sub-species of *Trypanosoma brucei*. *T. b. gambiense* accounts for approximately 95% of HAT infections and occurs across East and Central sub-Saharan Africa. The remaining infections are caused by *T. b. rhodesiense* in West and Southern Africa. The disease has two stages: Stage 1, where the parasites are limited to the bloodstream, interstitial fluids and lymph of the patient, and stage 2, where parasites are also found in the central nervous system.

**Figure 1:**
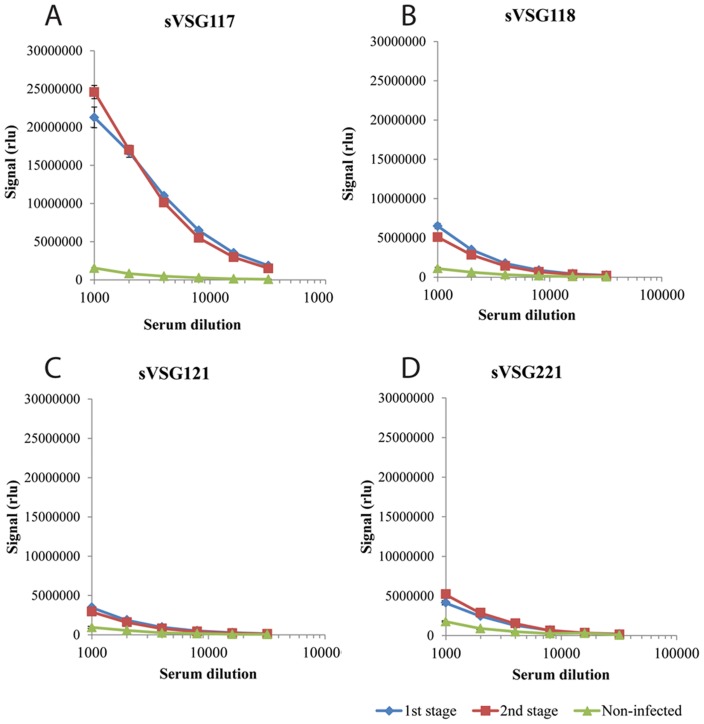
Soluble form VSG ELISA results with pooled human *T. b. gambiense* infection and control sera. Sera were pooled from 1^st^ (blue lines) and 2^nd^ (red lines) stage *T. b. gambiense* infected HAT patients and from non-infected patients (green lines) and titrated against ELISA plates coated with sVSG variants 117 (panel A), 118 (panel B), 121 (Panel C) and 221 (Panel D). Results are expressed as means ± 1 standard deviation of triplicate measurements. The y-axis is Relative Luminescence Units (RLU).

**Figure 2:**
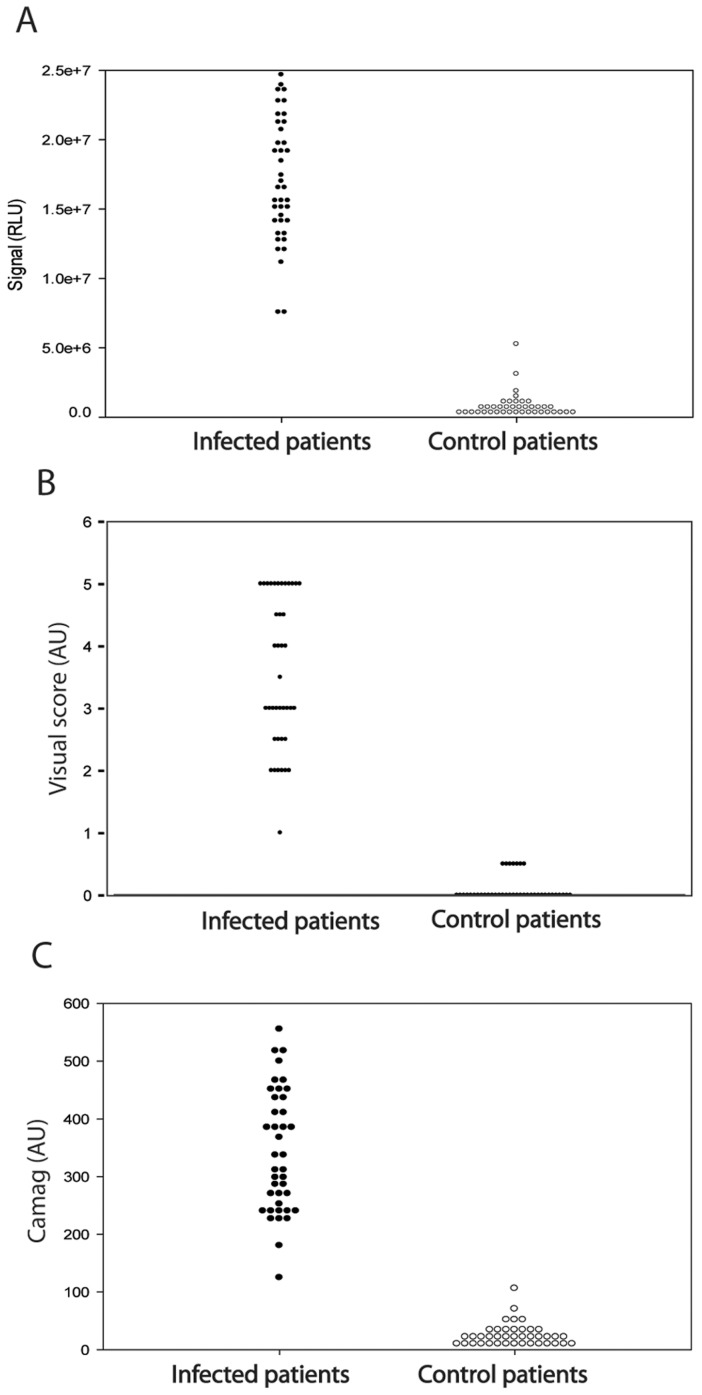
Scatter plots of the immunoreactivities of infection and control sera to sVSG117. In each plot, data from infection (left) and matched uninfected control (right) sera are plotted against (A) ELISA results, (B) single-antigen LFT results using visual scoring and (C) single-antigen LFT results using Camag laser densitometry scoring.

**Figure 3:**
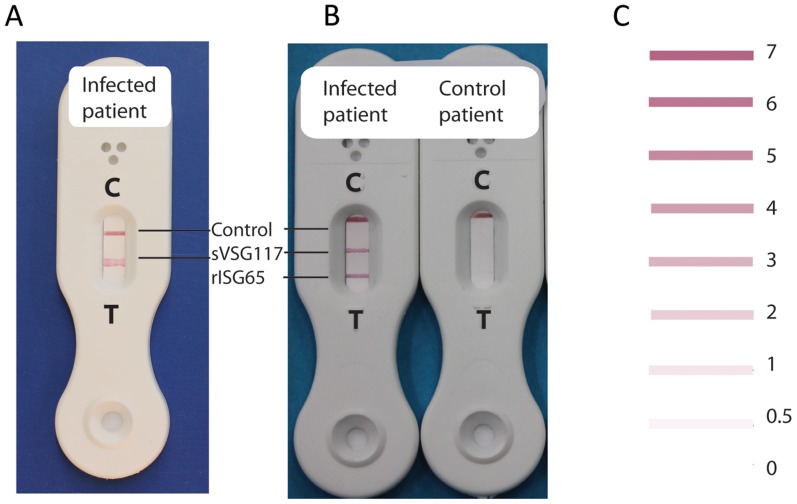
Single- and dual-antigen prototype LFTs. Photographs of (A) a single-antigen (sVSG117) prototype LFT developed with *T. b. gambiense* infection serum and (B) a dual-antigen prototype LFT developed with *T. b. gambiense* infection serum (left) and control uninfected serum (right). The positions of the ‘test complete’ control lines and antigen test lines are as indicated. The band intensities are scored visually by comparison with a test card (C).

In recent years, the official number of recorded HAT cases has fallen below 10,000 per year, although possible under-reporting suggests that this is likely a minimum figure [1]–[3]. Nevertheless, with new therapeutic regimes [4]–[6] and with a repurposed drug (fexinidazole) [7] and a new chemical entity (an oxaborole) [8] in clinical trials, the potential to eliminate HAT from many regions of sub-Saharan Africa at last exists. However, disease elimination requires excellent and convenient field diagnostics. Currently, the diagnosis of infected individuals relies principally on screening teams that visit at-risk communities and from patients seeking medical help [9], [10]. Some patients with *T. b gambiense* infections remain asymptomatic for years, so early diagnosis of infected individuals benefits not only the patient but also the community where these individuals can act as parasite reservoirs [11]. The most widely used diagnostic for suspected *T. b. gambiense* infections is the Card Agglutination Test for Trypanosomiasis (CATT). This serological test detects host antibodies to a suspension of fixed and stained *T. b. gambiense* trypanosomes expressing variant surface glycoprotein (VSG) variant LiTaT1.3 [12]. Over the years, the CATT screening tool has been optimised to improve stability, sensitivity (ranging from 87% to 98%) and specificity, (95%) and thermostability [1], [13]–[16]. A positive CATT is followed up by microscopic examination of blood buffy coat smears. Until recently, stage 1 and stage 2 treatment regimes were different, and the latter much more toxic, such that positive diagnosis of infection was then staged by microscopic examination of Cerebral Spinal Fluid (CSF) for the presence of trypanosomes and/or lymphocytes. However, the use of nifurtimox and eflornithine combination therapy (NECT) [4]–[6] in recent years has largely removed the need for staging diagnosis in *T. b gambiese* infections.

Despite its usefulness, the CATT screening tool has several widely acknowledged limitations [17]–[20]. It requires cultivation of infectious parasites for its manufacture, trained personnel for use and the read out is subjective, causing variability in reported sensitivity and specificity [16], [21]. Significantly, some *T. b. gambiense* strains do not express the LiTat1.3 VSG gene and, therefore, patients infected with these strains do not generate detectable antibodies [22]. For the same reason, the CATT test cannot detect *T. b. rhodesiense* infections [23].

There are challenges to developing improved diagnostic assays and devices for HAT. Due to the very low parasite levels in patients infected with *T. b. gambiense*, a test that detects host antibodies (rather than parasite antigens) is considered more likely to have the necessary sensitivity. The WHO recommends that point of care tests (POCT) should follow the ‘ASSURED’ criteria; which states that a POCT device should be affordable, sensitive, specific, user-friendly, rapid, equipment-free and deliverable to the people at need. Lateral flow tests (LFTs) are inexpensive and simple devices that can rapidly detect nanogram amounts of antibodies in finger-prick blood samples without the need for any ancillary equipment [24]. A first-generation LFT for *T. b. gambiense* infections, that uses two different purified native VSG antigen bands (LiTat1.3 and LiTat1.5) to detect anti-VSG antibodies, has recently entered clinical use as CATT replacement [25]. We have also produced a promising prototype LFT using a recombinant invariant surface glycoprotein (rISG65) antigen [26]. In this paper, we identify another soluble form VSG (sVSG117 also known as sVSG MITat1.4) with excellent diagnostic properties that we have used together with rISG65 to create a prototype dual-antigen LFT that detects *T. b. gambiense* infections and, to some extent, *T. b. rhodesiense* infections.

## Materials and Methods

### Ethics statement

The serum samples used in this study were from the WHO HAT Specimen Biobank, archived at the Pasteur Institute, Paris. Patients were recruited by WHO to provide serum samples as described in [27] for the development of new diagnostic tests for HAT and patient consent was collected by WHO at the time of sample collection. Further local ethical approval for this study was granted by the Tayside Ethics Review Board. Rodents were used to propagate *T. brucei* parasites for the purification of soluble form variant surface glycoproteins (sVSGs). The animal procedures were carried out according the United Kingdom Animals (Scientific Procedures) Act 1986 and according to specific protocols approved by The University of Dundee Ethics Committee and as defined and approved in the UK Home Office Project License PPL 60/3836 held by Michael A.J. Ferguson.

### Patient sera

All patients were tested with the CATT test (which was followed by parasitological analysis) and examined for clinical symptoms of HAT [27]. Serum samples were stored in the WHO HAT Specimen Biobank at −80°C and shipped to Dundee on dry ice where they were thawed, divided into aliquots and stored at −20°C.

### sVSG preparations

Bloodstream form *T. b. brucei* Lister strain 427 clones expressing four different VSG variants (117, 118, 121 and 221) were cultivated in rodents as described in [28] and sVSGs were purified by a simplified version of the method of Cross [28], as described in [29]. The sVSGs were further purified by gel-filtration using a Sephacryl-S200 column (4×90 cm) equilibrated and eluted with 0.1 M NH_4_HCO_3_. The gel-filtration purified sVSGs were lyophilised to remove NH_4_HCO_3_ and stored as dry powders at 4°C before use. Samples were run on an SDS-PAGE gel to check for purity and were considered >95% pure (data not shown).

### Enzyme-linked immunosorbent assays (ELISA)

The ELISA plate preparation details and protocols were as described in [26]. ELISAs were carried out on both pooled and individual serum samples. The pooled sera were from stage 1 *T. b. gambiense* patients (n = 10), stage 2 *T. b. gambiense* patients (n = 40) and matched uninfected patient sera (n = 50). Pooled sera were diluted 1∶1000 in in phosphate buffered saline containing 0.1% w/v bovine serum albumin (PBS/BSA) and plated in triplicate in serial (doubling) dilutions in PBS/BSA to 1∶32000. Individual sera were diluted to 1∶1000 in PBS/BSA and applied to ELISA plates in triplicate.

### Randomisation and coding of sera

For the sVSG117 single antigen lateral flow test pilot study, forty *T. b. gambiense* infection sera and forty matched uninfected control sera were randomised and coded by a member of the University of Dundee Tissue Bank. For the dual-antigen lateral flow test virtual field trial, 431 serum samples, representing a mixture of *T. b. gambiense* (n = 150) and *T. b. rhodesiense* (n = 56) infection sera and matched uninfected control sera (n = 150 for *T. b. gambiense* and n = 75 for *T. b. rhodesiense*) were randomised and coded by the WHO HAT specimen Biobank.

### Lateral flow test prototype production and use

We supplied BBI Solutions with 5 mg sVSG117 to make single antigen sVSG117 LFT prototype devices for preliminary studies and with a further 7 mg of sVSG117 and 7 mg of rISG65 [26] to make dual-antigen LFT prototypes. BBI Solutions is an inmmunoassay development and manufacturing company that has completed more than 250 lateral flow projects over the last 25 years, with manufacturing sites in Europe, USA and South Africa. Both serum- and blood-accepting pad devices were made. For LFTs without blood pads, aliquots of 5 µl of patient sera diluted with 15 µl of PBS were added to the LFTs followed by an 80 µl of chase-buffer (PBS containing 0.05% Tween 20). For LFTs with blood pads, aliquots of 5 µl of patient serum were mixed with 5 µl PBS and 10 µl of freshly reconstituted human type-O blood erythrocytes. These mixtures were added to the LFTs, followed by 80 µl of chase-buffer (PBS containing 0.05% Tween 20). Tests were discarded if upper control line was not clearly visible. After 30 min, scoring of the test bands was performed by visual comparison of freshly completed tests with a scoring card. For the virtual field trial, two people scored all of the LFT devices independently. If there was disagreement about the infection-status of a given serum sample, a third individual provided adjudication.

### Statistics

Line graphs were generated by Microsoft Excel. Receiver Operator Characteristic (ROC) curves, antigen scatter plots and tables of sensitivity and specificity scores were generated by SigmaPlot 12.

## Results

### Identification of sVSG117 as a potential diagnostic antigen

Our original rationale for testing HAT sera against a panel of different sVSGs was to look for the presence of anti-Cross Reacting Determinant (CRD) IgG antibodies. The CRD is a peptide-independent epitope common to all sVSGs that is created upon the cleavage of VSG glycosylphosphatidylinositol (GPI) membrane anchors by GPI-specific phospholipase C (GPI-PLC) during cell lysis [30]. However, the ELISA data showed that while there was anti-peptide and/or anti-CRD IgG antibody titre to all four sVSGs, the immunoreactivity of both stage 1 and stage 2 *T. b. gambiense* HAT patient sera to sVSG117 was far higher than to the other three (Figure 1). From this result, we decided to pursue sVSG117 as a potential diagnostic antigen in its own right. We therefore proceeded to screen randomised and coded sera from 40 *T. b. gambiense* infected patients and 40 matched uninfected control patients against sVSG117 coated ELISA plates (Figure 2A). These data strongly suggested that immunoreactivity to sVSG117 might be used to reliably discriminate infection from control sera. Consequently, sVSG117 was developed into an un-optimised single-antigen prototype lateral flow test (Figure 3A), which was used with the same set of 80 randomised and coded serum samples. The visual test scores of the decoded data are shown in (Figure 2B). The bands were also assessed by quantitative laser densitometry, as described in [26], (Figure 2C) which showed an excellent correlation between visual- and densitometer-based scoring, with an r^2^ correlation value of 0.957. These data enabled us to set a cut-off threshold of ≥1 visual units for discriminating infected from uninfected sera on this LFT device. Using this threshold, the test appeared to have 100% sensitivity and 100% sensitivity, albeit based on a relatively small sample set.

### Preliminary evaluation of a dual-antigen lateral flow test prototype

An un-optimised dual-antigen lateral flow test prototype, containing one band of recombinant antigen rISG65-1, previously described in [26], and one band of the native sVSG117 antigen, described here, was manufactured by BBI Solutions (Figure 3B). The dual-antigen LFTs were manufactured using the same antigen coupling conditions as the individual rISG65 [26] and sVSG117 (this study) single-antigen LFTs. Thus, visual score cut-offs of ≥2 for the rISG65 band [26] and of ≥1 visual units for the sVSG117 band were expected to define positive immunoreactivity to each antigen, respectively. However, to establish visual cut off values directly for this new LFT, the same 80 randomised serum samples described above were tested blind with the dual-antigen LFT and scored. After decoding, cut-offs were confirmed as being ≥2 and ≥1 for the rISG65 and sVSG117 test lines, respectively. Using these values, and the criterion of two positive test lines to define an infection, the device gave 100% sensitivity and 97.5% specificity in this pilot study with a limited number of serum samples (n = 80).

### Virtual field trial of the dual antigen lateral flow test

**Figure 4:**
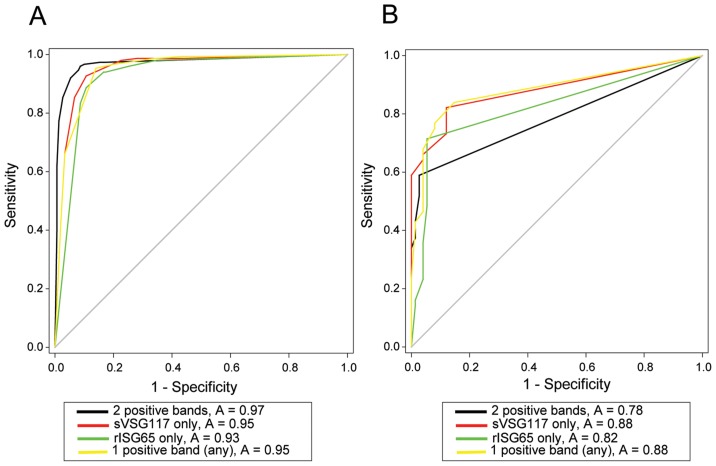
Receiver Operating Characteristics (ROC) graphs of LFT performance. Following independent decoding of the *T. b. gambiense* (A) and *T. b. rhodesiense* (B) patient serum samples, the data were plotted as cumulative sensitivity (true positive rate) versus specificity (false positive rate). The greater the area under the ROC curve, the greater the accuracy of the test. The curves in black are for ‘both bands positive = infected’; the curves in red are for ‘sVSG117 band positive = infected’; the curves in green are for ‘srISG65 band positive = infected’ and the curves in yellow are for ‘any band positive = infected’.

A virtual field study was performed to assess the diagnostic potential of the dual-antigen LFT. First, aliquots of 431 randomized and coded serum samples, provided by the WHO HAT Specimen Biobank, were mixed with an aliquot of human type-O erythrocytes, provided by the Tayside blood-bank, to produce pseudo blood samples containing red blood cells as well as serum antibodies. These samples were added to the LFTs fitted with blood pads, followed by chase buffer, and read independently by two individuals after 30 min. The LFT was deemed to be positive if the rISG65 band and sVSG117 had mean visual scores of ≥2 and ≥1, respectively. After decoding by colleagues at the WHO HAT Specimen Biobank, we were able to plot ROC curves (Figure 4) and separately assess the sensitivity and specificity of the LFT to detect *T. b. gambiense* and *T. b. rhodesiense* infections using the following criteria: (i) two positive antigen bands = infection, (ii) a positive sVSG117 band = infection, (iii) a positive rISG65 band = infection and (iv) any one positive antigen band = infection. The results, in terms of sensitivity, specificity and the respective 95% confidence intervals (CI) are summarised in (Table 1).

**Table 1:** Sensitivity, specificity and the respective 95% confidence intervals (CI) using different LFT criteria for *T. b. gambiense* and *T. b. rhodesiense* infections.

Infectious species	LFT criterion of a positive test	Sensitivity	95% CI	Specificity	95% CI
*T. b. gambiense*	Two positive test lines	97.3%	93.3 to 99.2	83.3%	76.4 to 88.9
*T. b. gambiense*	Minimum of one positive test line	99.3%	96.3 to 99.9	46.0%	37.8 to 54.3
*T. b. gambiense*	sVSG117 positive test line only	98.7%	95.3 to 99.8	64.0%	55.8 to 77.0
*T. b. gambiense*	rISG65-1 positive test line only	98%	94.3 to 99.6	65.3%	57.1 to 72.9
*T. b. rhodesiense*	Two positive test lines	58.9%	44.9 to 71.9	97.3%	90.7 to 99.7
*T. b. rhodesiense*	Minimum of one positive test line	83.9%	71.7 to 92.4	85.3%	75.3 to 92.4
*T. b. rhodesiense*	sVSG117 positive test line only	73.2%	59.7 to 84	88 .0%	78.4 to 94.4
*T. b. rhodesiense*	rISG65-1 positive test line only	69.6%	55.9 to 81.2	94.7%	86.7 to 98.5

## Discussion

Although we selected sVSG117 as a potential diagnostic antigen from empirical data in this study, our results are also consistent with population genetics studies that show that the gene encoding this VSG variant (the same as VSG AnTat 1.8) is ubiquitous in *T. b. gambiense* isolates [31], [32], whereas those for VSGs 121 and 221 are not [33].

In the virtual field trial, using two positive antigen bands as the criterion for infection, the sVSG117 and rISG65 dual-antigen lateral flow test prototype showed a sensitivity of 97.3% (95% CI: 93.3 to 99.2) and a specificity of 83.3% (95% CI: 76.4 to 88.9) for the detection of *T. b. gambiense* infections. The sensitivity is comparable to those reported (87–100%) for the currently deployed CATT test and for the latex agglutination test which uses diluted blood and latex beads coated with three different VSG variants (LiTat1.3, 1.5 and 1.6) [17], but is poorer with respect to specificity, which have been reported as 85–97% for CATT and 96–99% for the latex test [13]–[17]. Nevertheless, the dual-antigen LFT described here is only a prototype that needs to undergo extensive optimization with respect to antigen-gold coupling, antigen loading of the test lines and composition of the chase buffer. We therefore suggest that one or both of the sVSG117 and rISG65 antigens be seriously considered for use in the next generation of clinical LFT devices for the diagnosis of *T. b. gambiense* HAT.

We note that for our prototype dual-antigen LFT the specificity performance of each individual antigen is relatively poor for detecting *T. b. gambiense* infections (Table 1). For example, the rISG65 test line shows a sensitivity of 98.0% (95% CI: 94.3 to 99.6) but a specificity of only 65.3% (95% CI: 57.1 to 72.9). We have previously reported the sensitivity and specificity performance of a visually-read single-antigen LFT using rISG65 as 88% (95% CI: 73 to 96) and 93% (95% CI: 80 to 98), respectively [26]. While there is good overlap between the 95% confidence intervals for these two assessments with respect to sensitivity, we note that there is a discrepancy with respect to specificity. However, the previous assessment [26] only used 80 randomized infection and control serum samples and we suggest that the figures reported in (Table 1) are likely to be more accurate given the significantly greater sample size (and wider geographic sampling) used in the virtual field trial. Another possibility is that some of the ‘false-positive’ results, which drive down the specificity figures for the dual-antigen LFT, might be due to asymptomatic true positives that had been missed by the CATT test in the virtual field trial cohort. As previously noted, this is entirely possible as not all *T. b. gambiense* strains express the LiTat1.3 VSG upon which the CATT test is based [22].

The dual-antigen LFT did not perform as well for detecting *T. b. rhodesiense* infections using two positive antigen bands as the criterion for infection, with a sensitivity of only 58.9% (95% CI: 44.9 to 71.9) and specificity of 97.3% (95% CI: 90.7 to 99.7). A potentially confounding issue for *T. b. rhodesiense* immunodiagnosis is the typically acute nature of these infections compared to typically chronic *T. b gambiense* infections, with the latter more likely to produce robust antibody responses to parasite antigens. However, using any one (or both) positive antigen band(s) as the criterion for *T. b. rhodesiense* infection improved the sensitivity to 83.9% (95% CI: 71.7 to 92.4) with a specificity of 85.3% (95% CI: 75.3 to 92.4). As of yet there have been no confirmed cases of co-existing *T. b. rhodesiense* and *T. b. gambiense* infections [34], and given the current lack of immunodiagnostics for *T. b. rhodesiense* infections [35], an optimized version of the dual-band LFT using the relaxed criteria of one or two positive band(s) to diagnose HAT might be clinically useful in *T. b rhodesiense* endemic regions.

Taken together, the results described in this paper encourage further development of the dual-antigen LFT device described here (or one or both of its antigens, *i.e.*, recombinant rISG65-1 and native sVSG117) for clinical use for the detection of *T b. gambiense* infections and, possibly, for *T. b rhodesiense* infections. LFT technology offers advantages over CATT with respect to the “affordable, user-friendly, rapid, equipment-free and deliverable to the people at need” components of the WHO ‘ASSURED’ criteria of “affordable, sensitive, specific, user-friendly, rapid, equipment-free and deliverable to the people at need”. However, the “sensitive” and “specific” components of the criteria are clearly also key to success and, while data on the currently deployed first-generation LFT [25] (that uses native sVSGs LiTat1.3 and LiTata1.5) are yet to be published, the FIND web site suggests that its performance is comparable to CATT. Like the CATT test, and the currently deployed LFT [25], our dual-antigen LFT requires the cultivation of parasites to make the native sVSG117 component, although sVSG117 can at least be prepared from non-human infectious *T. b. brucei*. Nevertheless, the ideal second-generation LFT is likely to use two recombinant, rather than native, antigens and recombinant ISG65 [26] and/or VSG domains could be the answer.
